# 

**Published:** 1888-04-21

**Authors:** 


					?"PRICE ONE PENNY.
-i?o. 82,vVol. IV-
-April 21st, 1888.
(Registered at the General Post Office
as a Newspaper.]
H ?
Per Annum, 6s. 6d? post free.
Monthly Parts, 6d.; post free, 7id.
Ditto per Ann.i 7s. 6d. post free.
A Weekly Institutional Journal of
Scicnct, /Ifotiritira, IRumttg, attir flbljtlantljropg.

				

